# Nodular Fasciitis of the Parotid Gland Mimicking Pleomorphic Adenoma: Cytological Features of a Diagnostic Pitfall

**DOI:** 10.1002/dc.70151

**Published:** 2026-06-01

**Authors:** Xiaobing Jin, Steven Hrycaj, Stephanie L. Skala, Jonathan B. McHugh, Richard L. Cantley

**Affiliations:** ^1^ Department of Pathology University of Michigan‐Michigan Medicine Ann Arbor Michigan USA

## Abstract

Nodular fasciitis (NF) is a self‐limiting mesenchymal neoplasm that typically occurs in the subcutaneous tissue of the extremities, trunk, and head and neck region; involvement of the parotid gland is very rare. The cytological features of NF can closely mimic those seen in salivary gland neoplasms, particularly pleomorphic adenoma (PA), potentially leading to misdiagnosis. We present the case of a 19‐year‐old female with a parotid gland mass, initially diagnosed as a salivary gland neoplasm on fine needle aspiration (FNA), but ultimately confirmed to be NF following thorough examination of the surgically resected specimen. Distinguishing NF from PA and other primary salivary gland neoplasms is crucial for appropriate management. A more definitive diagnosis of NF can be established on cytological material using ancillary studies, particularly in situ hybridization (ISH) analysis for *USP6* rearrangement, thereby guiding more appropriate clinical management. This report aims to highlight this important diagnostic pitfall and alert cytopathologists by providing an illustrative example.

## 1 Introduction

Nodular fasciitis is a benign, self‐limiting myofibroblast proliferation typically originating in subcutaneous tissue, most frequently affecting young adults and commonly arising in the extremities, trunk, and head and neck region [1]. NF involving the parotid gland is exceedingly rare and can pose a significant diagnostic challenge, as it may be misinterpreted as benign or malignant salivary gland neoplasms [2, 3, 4]. We report a case of a parotid mass initially diagnosed as a salivary gland neoplasm favoring PA, which was ultimately confirmed as NF upon histological examination.

## 2 Clinical Presentation

A 19‐year‐old female patient presented with a right neck mass first noted approximately two months prior. She did not recall any history of trauma. The mass had initially been growing slowly but more recently underwent rapid growth, with the patient estimating that it had doubled in size in recent weeks. She reported localized pain, as well as shooting pain radiating to her right ear.

**FIGURE 1:**
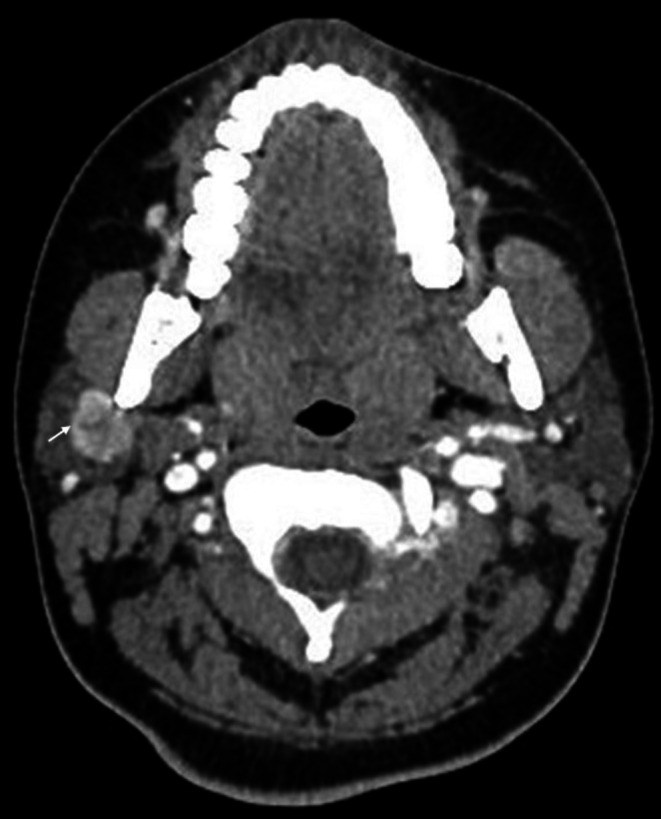
CT imaging revealed a heterogeneously enhancing lesion (arrow) involving the right parotid gland.

CT imaging (Figure 1) revealed a nodular, heterogeneously enhancing lesion involving the superficial lobe of the right parotid gland, located immediately posterior to the mandibular angle. The mass measured 1.8 × 1.5 × 1.5 cm. The imaging characteristics were concerning for a primary parotid neoplasm.

**FIGURE 2:**
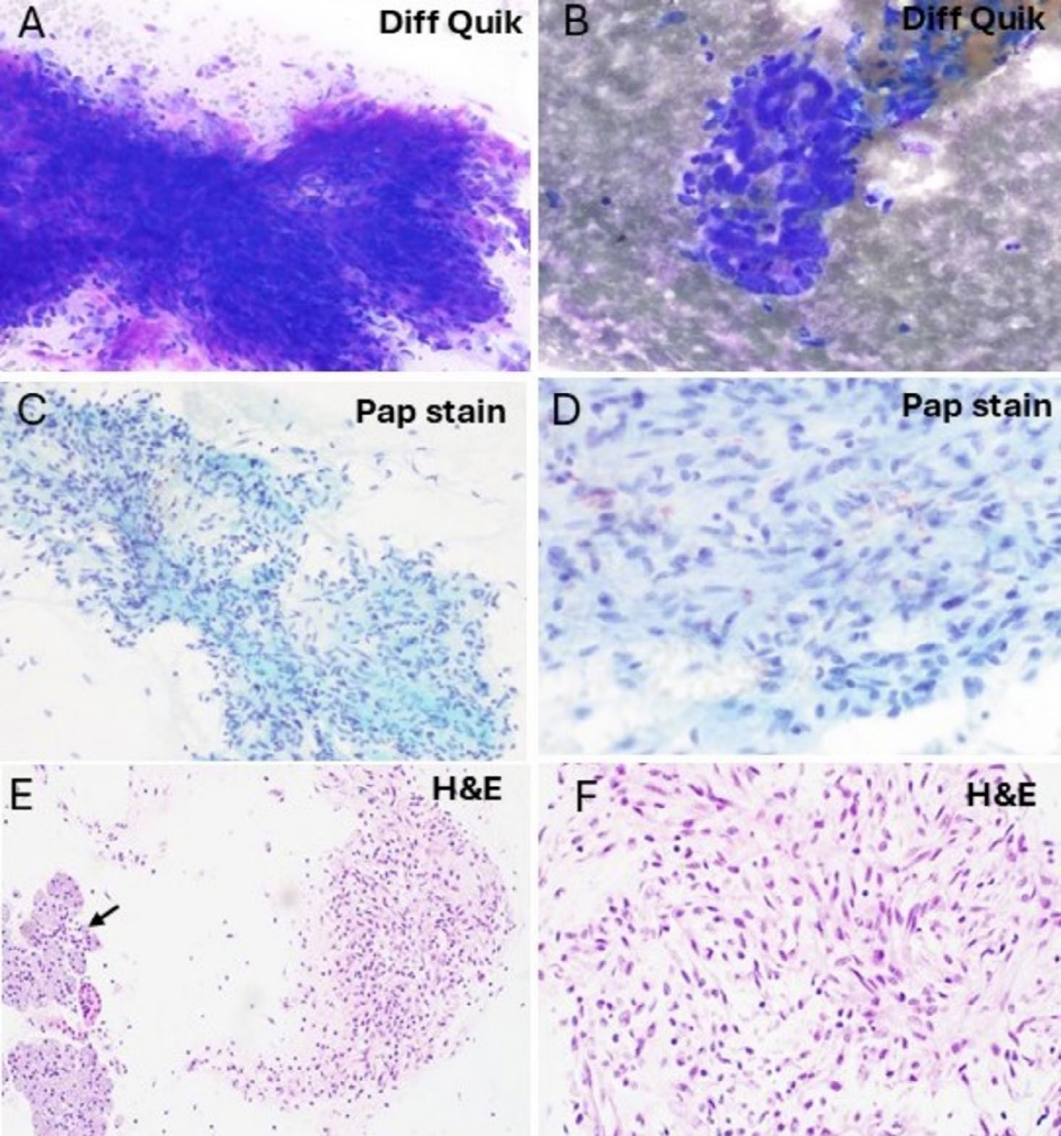
FNA of the right parotid mass. Diff‐Quik stained smear slides show spindled and polygonal cells within wispy matrix material (A, ×200). Rare clusters of bland epithelioid cells form vague ductal architecture (B, ×400). Similar cells are observed in Papanicolaou‐stained (Pap) slides, with background containing scattered lymphocytes, neutrophils, scattered red blood cells (C, ×200; D, ×400). Hematoxylin and Eosin (H&E) staining of cell block material reveals similar spindle cells with inflammatory cells (E, ×200; F, ×400) as well as normal acini (E, ×200, arrow).

FNA was performed, and the initial rapid on‐site evaluation diagnosed as “neoplastic cells present.” Smear slides and cell block revealed a neoplastic population of spindle‐shaped and polygonal myoepithelial‐like cells embedded within wispy matrix material (Figure 2A). The nuclei of these cells were elongated and oval shaped with inconspicuous nucleoli. Rare clusters of bland epithelioid cells forming vague ductal architecture were identified (Figure 2B). Similar cells are observed in Papanicolaou‐stained (Pap) slides, with background containing scattered lymphocytes, neutrophils, and red blood cells (Figure 2C,D). Hematoxylin and Eosin (H&E) staining of cell block material reveals similar spindle cells with inflammatory cells (Figure 2E,F) as well as normal acini. A diagnosis of “Positive for neoplastic cells” was rendered. The differential diagnosis provided in a comment focused on salivary neoplasms with myoepithelial differentiation and included cellular pleomorphic adenoma, myoepithelioma, and low‐grade salivary gland malignancies.

**FIGURE 3:**
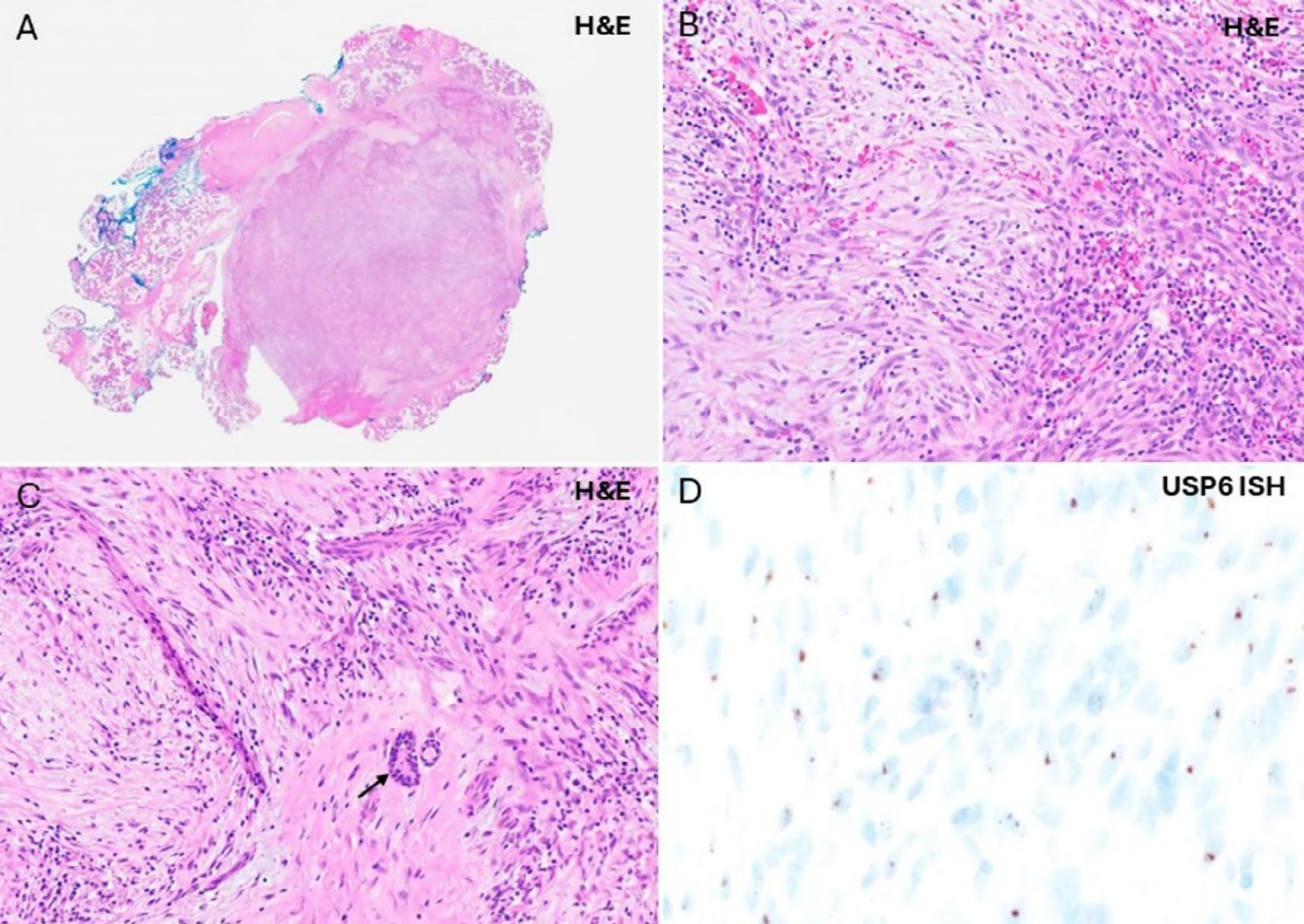
H&E staining of the surgical specimen reveals a cellular spindle cell proliferation displaying myxoid and tissue culture‐like appearance (A, ×0.5; B, 200×). Lymphocytes and extravasated erythrocytes are present (B, ×200). Focal embedded ductal components are identified (C, ×200). *USP6* RNA chromogenic in situ hybridization (ISH) is positive (D, 1600×).

Given the clinical and radiological concern, coupled with cytological findings suggestive of a salivary gland neoplasm, the patient underwent right total parotidectomy and excision of right deep cervical lymph nodes. Histological examination revealed a cellular spindle cell proliferation displaying a myxoid and tissue culture‐like appearance (Figure 3A). Lymphocytes and extravasated erythrocytes were present (Figure 3B). Focal embedded salivary ductal elements were identified (Figure 3C). No significant nuclear hyperchromatic or pleomorphism was observed, and mitotic figures were rare, supporting the diagnosis. The final diagnosis was nodular fasciitis, involving the deep lobe of the right parotid gland. All submitted lymph nodes were negative for neoplasm.

## 3 Discussion

The cytological features of NF in the parotid gland have been described in only a limited number of studies [5, 6, 7, 8]. In a multi‐institutional case series [7], 15 cases of NF involving the parotid gland were reviewed. All cases were composed of predominantly spindle cells, with the majority of them displaying a tissue‐culture appearance (69.2%) and at least some component of myxoid stroma (76.9%). Inflammatory cells were present in a minority of the cases (23.1%). One case (7.7%) revealed background salivary gland tissue. Immunohistochemical stains were performed on four cases and showed negativity for S100 and cytokeratin. Ultimately, nine cases were classified descriptively as spindle cell lesion or spindle cell neoplasm, while three were classified as salivary gland neoplasm, including two favoring a diagnosis of cellular pleomorphic adenoma. One case was diagnosed as “probable NF” and only a single case was definitively diagnosed as NF. This case series highlights the challenges in achieving definitive cytological diagnosis of NF in the parotid gland, primarily due to the non‐specific and overlapping cytomorphological features with other entities. The diagnosis of NF often requires a combination of clinical and radiological findings, cytological features and pertinent negative results to exclude certain entities in the differential diagnoses.

**FIGURE 4:**
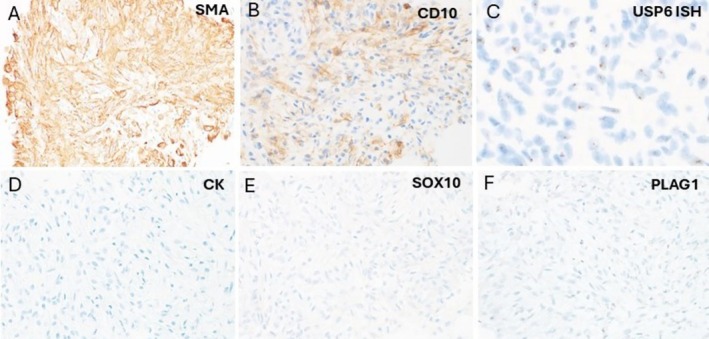
Immunohistochemical (IHC) staining demonstrates that the spindle cells are positive for SMA (A, ×400) and CD10 (patchy) (B, ×400). *USP6* RNA chromogenic in situ hybridization (ISH) is positive (C, ×1600). Cytokeratin cocktail (CK) (D, ×400), SOX10 (E, 400×), and PLAG1 (F, 400×) are negative.

Silvanto [9] and Peng [8] each reported an FNA cytology case of the parotid gland in which NF was initially misdiagnosed as PA. In both cases, the cytology revealed aggregates of spindle cells embedded in a fibrillary or mucoid stroma with scattered plasmacytoid cells also observed. Saad [5] described two parotid aspirates showing spindle cells within mucoid and fibrillar background, along with rare ganglion cell‐like cells exhibiting polyhedral or triangular shapes; these findings were initially interpreted as PA, but NF was confirmed on subsequent surgical specimen. Consistent with these reports, our case shared similar cytomorphological features including spindle cells and fibrillary stromal material. However, the more specific features of PA—such as chondromyxoid matrix, plasmacytoid myoepithelial cells, and a neoplastic epithelial population—were absent in our case and in previous reports. Normal salivary gland tissue, including acini and ducts, may be presented in the aspiration [7] due to the sampling of background salivary parenchyma. Distinction of normal salivary duct elements and the epithelial component of biphasic salivary gland tumors can be difficult as both have a bland, basaloid appearance. However, when epithelial elements are scant, such as in our case, care should be taken to avoid overinterpreting these as neoplastic. Notably, in this and previously reported cases, the diagnosis of PA or salivary gland neoplasm was based solely on cytomorphological features. No immunohistochemical stains were performed to confirm the myoepithelial or epithelial origin of the lesional cells. Retrospective immunohistochemical staining of cell block material in our case demonstrated that those spindle cells were positive for SMA (Figure 4A), suggesting myofibroblast differentiation, and showed patchy positivity for CD10 (Figure 4B). The spindle cells were negative for cytokeratin cocktail (Figure 4D), SOX10 (Figure 4E), and PLAG1 (Figure 4F). Although not entirely specific, this immunoprofile helps to rule out PA and other common epithelial‐myoepithelial salivary neoplasms and is compatible with fibroblastic/myofibroblast neoplasms such as NF. NF has been reported to harbor *USP6* fusion with various partners, most commonly *MYH9* [10]. While not widely available, fluorescence in situ hybridization (FISH) test for *MYH9‐USP6* arrangement [11] can provide a more definitive diagnosis. However, FISH analysis has a turnaround time of several days. *USP6* RNA in situ hybridization (ISH) is faster and less expensive and, with a reported 88% sensitivity and 100% specificity for aiding in the diagnosis of NF [12]. In our case, a retrospective *USP6* ISH analysis was positive in both cytology cell block material (Figure 4C) and resection specimen (Figure 3D). In cytology cases with features suspicious for NF, a positive *USP6* ISH result may be considered as confirmatory for *USP6* rearrangement and can support a definitive diagnosis. Comparison of cytomorphological features and ancillary studies between NF and PA is summarized in Table 1.

**TABLE 1:** Comparison of cytological features: nodular fasciitis versus pleomorphic adenoma.

	Nodular fasciitis	Pleomorphic adenoma
Cell arrangement	Predominanlty loosely cohesive fragments and single cells	Cohesive clusters and sheets, biphasic (epithelial and myoepithelial)
Cell morphology	Monomorphic spindle cells with unipolar and/or bipolar cytoplasmic processes; Ganglion‐like cells maybe present	Plasmacytoid/spindled myoepithelial cells and epithelial duct cells
Stromal matrix	Myxoid stroma, can also appear metachromatic on Diff‐Quik; lacks true chondroid differentiation	Fibrillary chondromyxoid stroma (Magenta‐red on Diff‐Quik)
Background	Myxoid; may contain inflammatory cells and extravasated red blood cells	Clean or myxoid; Inflammatory cells are rare
IHC (cell block)	Positive: Cytokeratin, SOX10,S100, P63+, PLAG1	Positive: SMA+, CD10 (patchy).
Molecular	USP6 rearrangement	PLAG1 or HMG2 fusion

NF can be homogenously or heterogeneously enhancing on CT and MRI and may present as well‐defined or ill‐defined and solid or partly or completely cystic. It can also exhibit aggressive features [13]. Due to the nonspecific nature of these radiologic findings, a biopsy is often necessary to establish a definitive diagnosis. Retrospective review of our case showed that the patient had a typical clinical presentation for NF: a rapidly growing superficial neck mass in a young adult. Distinguishing NF from PA and other salivary gland neoplasms is important since NF can spontaneously resolve without intervention, particularly in relatively small lesions [14]. Therefore, when NF is suspected, a more conservative approach may be taken by the clinician.

In conclusion, our case report demonstrates that the cytological features of NF of parotid gland may closely mimic those of PA. This case highlights the importance of considering both clinical and cytological findings in the FNA of head and neck lesions. NF should be included in the differential diagnosis of lesions with spindle cell features, especially when clinical symptoms are suggestive. When specific characteristic features of salivary origin are not clearly evident on FNA cytology, a basic immunohistochemical staining panel on cell block material should be considered to differentiate salivary gland lesions with spindle cell features from low‐grade neoplasms of mesenchymal origin. *USP6* ISH performed on cytological material can confirm *USP6* rearrangement and support a definitive diagnosis. Communicating NF as a diagnostic consideration to the surgeon may prompt conservative management strategies—including short‐interval observation or limited excision—thereby avoiding the morbidity of extensive surgical resection for this benign, self‐limiting condition. Greater awareness of NF as a cytological mimic of PA in the parotid gland is crucial to preventing misdiagnosing and ensuring more appropriate management.

## Author Contributions

**Xiaobing Jin:** acquired, analyzed, interpreted data and resource materials, drafted the manuscript and contributed significant revisions on subsequent drafts, contributed to final approval of version to be published, and agrees to be accountable for all aspects of the work in ensuring questions related to accuracy or integrity of any part of the work. **Steven Hrycaj:** interpreted data and resource materials, contributed to draft and final approval of version to be published. **Stephanie L. Skala:** interpreted data and resource materials, contributed to draft and final approval of version to be published. **Jonathan B. McHugh:** interpreted data and resource materials, contributed to draft and final approval of version to be published. **Richard L. Cantley:** acquired, analyzed, interpreted data and resource materials, drafted the manuscript and contributed significant revisions on subsequent drafts, contributed to final approval of version to be published, and agrees to be accountable for all aspects of the work in ensuring questions related to accuracy or integrity of any part of the work.

## Funding

The authors have nothing to report.

## Conflicts of Interest

The authors declare no conflicts of interest.

## Data Availability

The data that support the findings of this study are available on request from the corresponding author. The data are not publicly available due to privacy or ethical restrictions.
